# Nonmotor symptoms and Parkinson disease in United States farmers and spouses

**DOI:** 10.1371/journal.pone.0185510

**Published:** 2017-09-27

**Authors:** Srishti Shrestha, Freya Kamel, David M. Umbach, Laura E. Beane Freeman, Stella Koutros, Michael Alavanja, Dale P. Sandler, Honglei Chen

**Affiliations:** 1 Epidemiology Branch, National Institute of Environmental Health Sciences, Research Triangle Park, North Carolina, United States of America; 2 Biostatistics and Computational Biology Branch, National Institute of Environmental Health Sciences, Research Triangle Park, North Carolina, United States of America; 3 Occupational and Environmental Epidemiology Branch, Division of Cancer Epidemiology and Genetics, National Cancer Institute, Bethesda, Maryland, United States of America; 4 Department of Epidemiology and Biostatistics, College of Human Medicine, Michigan State University, East Lansing, Michigan, United States of America; National Institue on Drug Abuse, UNITED STATES

## Abstract

**Objectives:**

Few studies have evaluated the presence of multiple nonmotor symptoms (NMS) in relation to Parkinson disease (PD). Therefore, we examined cross-sectional associations between individual and multiple NMS and PD in the Agricultural Health Study.

**Methods:**

20,473 male farmers and 16,259 female spouses provided information on six NMS (reduced sense of smell, dream-enacting behavior, daytime sleepiness, infrequent bowel movement, depression, and anxiety) in the cohort’s 2013–2015 follow-up survey. 191 men and 68 women reported physician-diagnosed PD. We estimated odds ratios (ORs) and 95% confidence intervals (CIs) using multivariable logistic regression models separately by sex.

**Results:**

NMS were each associated with PD, with the strongest association for reduced sense of smell in men and dream-enacting behavior in women. The number of NMS showed a strong dose-response relationship with PD, particularly in men. ORs were 5.5 (95% CI 3.4–8.8) for one, 17 (95% CI 10.4–28.0) for two, and 53.4 (95% CI 33.2–86.1) for three or more NMS in men; the corresponding ORs were 4.6 (95% CI 2.3–9.5), 6.7 (95% CI 2.9–15.6), and 23.6 (95% CI 10.7–52.4) in women (*P*_NMS-interaction-with-sex_ = 0.07).

**Conclusions:**

The number of NMS was associated with PD in a dose-response manner and the association appeared stronger in men than in women. These findings should be further investigated in population-based prospective studies.

## Introduction

Nonmotor symptoms (NMS) are common among patients with Parkinson disease (PD), and some may precede PD diagnosis by years [1]. Previous prospective studies have shown that NMS such as impaired olfaction [2], sleep disturbances [3, 4], constipation [5], depression [6], and anxiety [7] are each associated with a higher risk of PD. These observations, in general, are consistent with the Braak hypothesis that PD Lewy body pathogenesis impairs extranigral structures such as olfactory bulb and lower brain stem before affecting the substantia nigra [8, 9].

Most NMS are not specific to PD. Nonetheless, if these NMS do result from a common or similar underlying pathology that may eventually progress to PD [9], one might speculate that PD patients will develop multiple NMS before PD diagnosis. Despite the fairly large body of literature on individual NMS and PD [1], few studies have evaluated the presence of multiple or specific combinations of NMS in relation to PD—although doing so may facilitate our efforts to understand predictors of PD development [10]. Further, recent evidence suggests potential sex differences in NMS presentation among PD patients [11]. Yet, few studies have investigated potential sex differences in the NMS-PD relationship.

We assessed six NMS (reduced sense of smell, dream-enacting behavior, excessive daytime sleepiness, infrequent bowel movement/constipation, depression, and anxiety) in the most recent follow up of a large cohort of US farmers and their spouses. We herein report their cross-sectional associations with prevalent PD, individually and in combination, separately for men and women.

## Methods

### Study population

The Agricultural Health Study (AHS) is an ongoing prospective cohort of licensed private pesticide applicators (mostly farmers and hereafter so identified) and their spouses from Iowa and North Carolina [12]. Briefly, 52,394 farmers completed an enrollment questionnaire in 1993–1997 (Phase 1) that asked about farming activities including lifetime use of pesticides, socio-demographics, and medical history. In Phase 1, farmers were also given a questionnaire to be filled out by their spouses at home, and 32,345 spouses completed the questionnaire. Follow up interviews were conducted in 1999–2003 (Phase 2), 2005–2010 (Phase 3), and 2013–2015 (Phase 4). The current analyses were limited to 23,478 male and 18,058 female participants who completed the Phase 4 survey which collected information on six NMS previously associated with PD. Since most farmers were men, and spouses female, we excluded 1,359 female farmers and 219 male spouses because of small numbers and our interest in studying sex-specific relationships. We also excluded 2,509 men and 971 women who had proxy respondents because the proxy questionnaire did not ask about NMS. Further, we excluded participants who provided inconsistent answers to PD diagnosis across AHS surveys (for example, those who reported PD diagnosis in Phase 3 but denied it in Phase 4, 25 men and 13 women) or missing information on smoking, leaving 20,473 men and 16,259 women for the current analysis (S1 Fig). All participants implied informed consent by returning study questionnaires and participating in the telephone interviews. Institutional review boards at the National Institute of Environmental Health Sciences and the National Cancer Institute approved the study protocol.

### Outcome ascertainment

At all AHS surveys, participants were asked to report whether they had ever been diagnosed with PD by a physician and their age at diagnosis. Most self-reported cases from Phase 1 and Phase 2 were invited to participate in an AHS add-on study, the Farming and Movement Evaluation (FAME) Study [13]. In FAME, self-reported PD cases were confirmed by movement disorder specialists, using medical records and an in-home assessment; 84% of self-reported cases were confirmed. For self-reported cases who reported PD diagnosis after FAME, we are currently validating PD diagnosis by collecting and evaluating relevant information on PD diagnosis, symptoms and treatment from patients and their treating physicians. Of those for whom we have completed the evaluation (241 cases), 201 (83%) were confirmed to have PD. A similar approach has been successfully used in many other population-based prospective cohorts [14–16]. Of eligible Phase 4 participants, 203 men and 81 women reported a PD diagnosis in any of the four AHS surveys. After excluding self-reported cases whose PD diagnosis was refuted by movement disorder specialists in the FAME study or the ongoing diagnostic validation (7 men and 7 women) and those with missing information on covariates (5 men and 6 women), the final analytic sample included 191 men and 68 women with potential PD. Comparison group includes 20,282 men and 16,191 women who reported not having PD in any of the AHS surveys.

### Nonmotor symptoms

The AHS Phase 4 questionnaire asked about six NMS that often occur in prodromal PD (details in S1 Table). We considered participants to have impaired olfaction if they reported a loss or significantly decreased sense of smell. Dream enacting behavior was assessed with a one-item validated screening question for probable rapid eye movement sleep behavior disorder (RBD) that was designed for epidemiological surveys [17]. We considered participants to have excessive daytime sleepiness if they answered “6–7 days per week” to the question “How often do you feel sleepy most of the day?” A similar question has been used elsewhere [18]. We defined infrequent bowel movement as ≤ 3–4 bowel movements per week or ever use of medications to help with bowel movements. Others have used similar questions [18, 19]. Depression was assessed using the 2-item Patient Health Questionnaire (PHQ-2) [20] and by asking about current use of prescribed medications for depression. We defined depression as a PHQ-2 score ≥ 3 [20] or current use of antidepressants. We defined anxiety as a score ≥ 3 on the 2-item Generalized Anxiety Disorder (GAD-2) scale [21]. In addition, we counted the number of NMS, which ranged from 0 to 6.

### Statistical analysis

We conducted primary analyses separately for men and women. Bivariate associations among NMS, PD, and covariates were assessed using chi-square tests and Spearman correlation coefficients as appropriate. We used multivariable logistic regression models to assess associations between NMS and PD, and report odds ratio (ORs) and 95% confidence intervals (CIs), adjusted for age and smoking at enrollment. Further adjustment for state of residence, lifetime days of any pesticide use, alcohol consumption, education, and history of head injury did not materially change the estimates, and so we present results from simpler models. In the main analysis, we dichotomized each individual NMS as present/absent. We categorized the number of NMS into four categories (0/reference, 1, 2, and ≥ 3), combining the highest numbers of symptoms because of sparse data in women. We tested statistical interactions between sex and NMS by including a cross-product term in regression models. In interaction analyses, we modeled the number of NMS as a continuous variable reflecting its approximately linear relationship with PD.

We used polytomous logistic regression models to evaluate the associations of NMS with PD by median disease duration (≤ 5 and > 5 years). We also evaluated the frequency of individual symptoms in relation to PD whenever we had sufficient cases. We used AHS data releases AHSREL20150600, P1REL201209_00, P2REL20120900, P3REL20120900, and Final_06172015. All statistical tests were two-tailed with α = 0.05. Statistical analyses were performed using SAS version 9.3 (SAS Institute Inc., Cary, NC).

## Results

We identified a total of 191 PD cases in male farmers and 68 in female spouses. The age-specific prevalence of PD among male farmers was 0.08% for age 40–49 years, 0.29% for 50–59 years, 0.72% for 60–69 years, 1.58% for 70–79 years, and 1.98% for 80+ years, and the corresponding prevalence among female spouses were 0%, 0.16%, 0.30%, 0.89%, and 0.50% respectively. Compared with same sex participants without PD, both male and female PD cases were older, and male cases were more likely to be never smokers (Table 1).

**Table 1:** Baseline characteristics of participants of the Agricultural Health Study.

	Men			Women		
	No PD N = 20282	PD N = 191		No PD N = 16191	PD N = 68	
Characteristics	N	N	OR (95% CI)^a^	N	N	OR (95% CI)^a^
Age at enrollment^b^						
≤ 45 years	9947	40	Ref	7562	12	Ref
46–55 years	5646	61	2.7 (1.8, 4.0)	4860	24	3.1 (1.6, 6.2)
56–65 years (men only)	3836	70	4.5 (3.1, 6.7)	-	-	-
> 65 years (men only)	853	20	5.8 (3.4, 10.0)	-	-	-
> 55 years (women only)	-	-	-	3769	32	5.4 (2.8, 10.4)
Smoking status at enrollment^c^						
Never smoker	11507	122	Ref	12168	53	Ref
Former smoker (men only)	6314	56	0.6 (0.4, 0.9)	-	-	-
Current smoker (men only)	2461	13	0.5 (0.3, 0.9)	-	-	-
Ever smoker (women only)	-	-	-	4023	15	0.9 (0.5, 1.6)
State						
Iowa	14245	135	Ref	11861	51	Ref
North Carolina	6037	56	0.9 (0.6, 1.2)	4330	17	0.8 (0.5, 1.4)
Education^d^						
≤ High school graduate	10470	106	Ref	6177	33	Ref
1–3 years beyond high school	5250	40	1.0 (0.7, 1.5)	4533	23	1.2 (0.7, 2.1)
College graduate or more	4181	43	1.3 (0.9, 1.9)	3755	12	0.9 (0.4, 1.7)
History of head injury^e^						
No	16353	147	Ref	14423	59	Ref
Yes	3660	41	1.4 (1.0, 1.9)	1515	8	1.4 (0.6, 2.9)

Abbreviations: CI, Confidence interval; OR, Odds ratio; Ref, Reference.

^a^Odds ratio for covariates adjusted for age.

^b^Four age categories for men and three for women due to difference in age distribution.

^c^Three smoking categories for men and two for women due to small sample size.

^d^N missing = 383 for men, and 1726 for women.

^e^N missing = 272 for men and 254 for women.

### Individual NMS and PD by sex

As expected, the prevalence of each NMS was greater among individuals with PD than those without (Table 2). Among men, all NMS were positively associated with PD after adjusting for age and smoking; the ORs for PD ranged from 3.5 (95% CI 2.4–5.0) for depression to 12.1 (95% CI 8.9–16.3) for reduced sense of smell. These associations were somewhat attenuated after mutual adjustment for all individual symptoms. NMS were moderately correlated with each other. Correlation coefficients ranged from 0.06 to 0.35, with the correlation between depression and anxiety being the highest.

**Table 2:** Non-motor symptoms and Parkinson's disease in the Agricultural Health Study.

	Parkinson's (N(%))^a^		OR (95% CI)	OR (95% CI)
	No	Yes	Model 1^b^	Model 2^c^
Men				
Reduced sense of smell	1905 (10)	110 (59)	12.1 (8.9, 16.3)^d^	8.6 (6.2, 11.8)^f^
Dream-enacting behavior	1507 (8)	70 (37)	7.7 (5.7, 10.4)	4.3 (3.1, 6.0)
Infrequent bowel movement	3295 (17)	101 (53)	4.6 (3.4, 6.1)^e^	3.0 (2.1, 4.1)
Excessive daytime sleepiness	704 (4)	26 (14)	3.7 (2.4, 5.7)	1.8 (1.1, 2.9)
Depression	1347 (7)	39 (21)	3.5 (2.4, 5.0)	1.6 (1.0, 2.6)
Anxiety	730 (4)	27 (15)	4.3 (2.8, 6.5)	1.2 (0.7, 2.1)
Women				
Reduced sense of smell	972 (6)	22 (34)	6.7 (4.0, 11.3)^d^	3.6 (2.0, 6.7)^f^
Dream-enacting behavior	745 (5)	17 (26)	8.2 (4.7, 14.3)	4.7 (2.5, 9.0)
Infrequent bowel movement	5342 (34)	39 (58)	2.5 (1.6, 4.2)^e^	2.0 (1.2, 3.4)
Excessive daytime sleepiness	424 (3)	9 (14)	5.2 (2.6, 10.8)	3.2 (1.4, 7.3)
Depression	2048 (13)	17 (27)	2.6 (1.5, 4.6)	1.2 (0.6, 2.5)
Anxiety	703 (5)	6 (10)	2.2 (0.9, 5.1)	1.0 (0.4, 2.5)

Abbreviations: CI, Confidence interval; OR, Odds ratio.

^a^N(%) of individuals with non-motor symptoms among groups with and without Parkinson's disease.

^b^Model 1: adjusted for age (restricted quadratic splines with knots at 38, 46, and 55 years for men and continuous for women), and smoking status (current, former, and never smokers for men, and never and ever for women); each symptom in a separate model.

^c^Model 2: same covariate adjustments as model 1, all symptoms in the model simultaneously.

^d^P-value for the cross-product term with sex in models with reduced sense of smell (P_interaction_ = 0.06).

^e^P-value for the cross-product term with sex in models with infrequent bowel movement (P_interaction_ = 0.06).

^f^P-value for the cross-product term with sex in mutually adjusted model (P_interaction_ = 0.02).

Among women, all NMS except for anxiety were positively associated with PD after adjusting for age and smoking, with ORs ranging from 2.5 (95% CI 1.6–4.2) for infrequent bowel movement to 8.2 (95% CI 4.7–14.3) for dream-enacting behavior. After mutual adjustment for all individual symptoms, ORs were attenuated, and the association with depression was no longer significant. Spearman correlation coefficients among NMS ranged from 0.04 to 0.32, with the correlation between depression and anxiety being the highest.

In analyses of statistical interaction between individual NMS and sex, we found suggestive interaction between sex and reduced sense of smell and infrequent bowel movement (both P_interaction_ = 0.06). In the model that was simultaneously adjusted for other symptoms, the interaction for sex with reduced sense of smell was statistically significant (P_interaction_ = 0.02).

### NMS and PD by disease duration

For most symptoms, associations with PD appeared to be stronger for disease with longer duration (> 5 versus ≤ 5 years) (Table 3); however, formal statistical tests did not show significant difference in ORs by PD duration in either sex.

**Table 3:** Non-motor symptoms and Parkinson's disease by disease duration in the Agricultural Health Study.

	PD diagnosis ≤ 5 years		PD diagnosis > 5 years	
	N (%)^a^	OR (95% CI)^b^	N (%)^a^	OR (95% CI)^b^
Men				
Number of cases	103		84	
Reduced sense of smell	54 (54)	6.8 (4.5, 10.5)	55 (66)	11.7 (7.1, 19.3)
Dream-enacting behavior	34 (33)	3.9 (2.5, 6.1)	36 (43)	5.1 (3.1, 8.2)
Infrequent bowel movement	50 (49)	2.5 (1.6, 3.9)	49 (58)	3.7 (2.3, 6.1)
Excessive daytime sleepiness	9 (9)	1.1 (0.5, 2.4)	16 (20)	2.5 (1.3, 4.7)
Depression	16 (16)	1.2 (0.7, 2.4)	23 (28)	2.3 (1.3, 4.2)
Anxiety	13 (13)	1.3 (0.6, 2.7)	13 (16)	1.0 (0.5, 2.2)
Women				
Number of cases	38		29	
Reduced sense of smell	11 (30)	2.8 (1.2, 6.5)	11 (41)	5.3 (2.2, 13)
Dream-enacting behavior	11 (29)	6.1 (2.7, 14)	6 (22)	3.3 (1.2, 9.1)
Infrequent bowel movement	20 (53)	1.6 (0.8, 3.2)	19 (68)	3.1 (1.2, 7.7)
Excessive daytime sleepiness	3 (8)	2.3 (0.6, 8.4)	6 (23)	4.3 (1.4, 13.1)
Depression	6 (17)	0.7 (0.2, 2.0)	11 (39)	2.3 (0.9, 6.1)
Anxiety	3 (8)	1.2 (0.3, 4.7)	3 (12)	0.8 (0.2, 2.9)

Abbreviations: CI, Confidence interval; OR, Odds ratio; PD, Parkinson's disease.

^a^N(%) of Parkinson’s patients with the specific non-motor symptoms

^b^Adjusted for age (restricted quadratic splines with knots at 38, 46, and 55 years for men and continuous for women), smoking status (current, former, and never smokers for men and never and ever for women), and all other other symptoms.

Note: Duration information missing for N = 4 for men and N = 1 for women.

### Frequency of individual NMS and PD

In general, we observed dose-response relationships between a symptom’s frequency and PD (Table 4). Among men, the OR comparing the highest frequency category with having no symptom was 19.2 (95% CI 11.0–33.5) for dream-enacting behaviors, 5.8 (95% CI 3.5–9.7) for daytime sleepiness, and 7.2 (95% CI 3.1–17.2) for infrequent bowel movement. Notably, for dream-enacting behaviors and infrequent bowel movement, even the least frequent category was associated with higher odds of PD compared to the referent. Women showed a similar pattern of associations, but analyses were limited by smaller sample sizes.

**Table 4:** Associations between frequency of individual non-motor symptoms and PD in the Agricultural Health Study.

	Men			Women		
	PD (N(%))^a^		OR (95% CI)^b^	PD (N(%))^a^		OR (95% CI)^b^
	No (N = 20282)	Yes (N = 191)		No (N = 16191)	Yes (N = 68)	
Dream-enacting behavior						
How often have you acted out in dreams?						
No	18414 (94)	120 (67)	Ref	15175 (96)	49 (79)	
< 3 times in life	280 (1)	6 (3)	3.9 (1.7, 8.9)	170 (1)	3 (5)	-
< once per month	593 (3)	18 (10)	5.2 (3.1, 8.5)	235 (2)	2 (3)	-
1–3 per month	192 (1)	18 (10)	15.6 (9.2, 26.4)	78 (0.5)	5 (8)	-
≥ Once per week	139 (1)	17 (10)	19.2 (11.0, 33.5)	61 (0.4)	3 (5)	-
Infrequent bowel movement						
Medications for bowel movement						
No	17441 (87)	109 (58)	Ref	12121 (76)	40 (60)	Ref
Yes	2529 (13)	79 (42)	3.8 (2.8, 5.2)	3800 (24)	27 (40)	1.8 (1.1, 3.0)
Typically, how often do you have bowel movements?						
≥ 2 per day	5290 (26)	25 (13)	Ref	2948 (19)	9 (13)	Ref
Once per day	12307 (62)	103 (55)	1.6 (1.0, 2.4)	8653 (54)	23 (34)	0.8 (0.4, 1.8)
5–6 per week	1205 (6)	16 (8)	2.2 (1.2, 4.1)	1710 (11)	10 (15)	2.0 (0.8, 4.9)
3–4 per week	949 (5)	37 (20)	6.3 (3.7, 10.5)	2102 (13)	22 (33)	3.7 (1.7, 8.0)
< 3 per week	157 (1)	7 (4)	7.2 (3.1, 17.2)	482 (3)	3 (5)	2.3 (0.6, 8.7)
Excessive daytime sleepiness						
How often do you feel sleepy most of the day?						
Never	6753 (34)	40 (21)	Ref	5055 (32)	13 (20)	Ref
<1 day per month	4434 (22)	35 (19)	1.5 (1.0, 2.4)	3916 (25)	10 (16)	1.1 (0.5, 2.5)
1–3 day per month	4276 (22)	35 (19)	1.7 (1.1, 2.7)	3463 (22)	12 (19)	1.6 (0.7, 3.6)
1–2 days per week	2542 (13)	30 (16)	2.3 (1.4, 3.7)	2055 (13)	9 (14)	1.8 (0.8, 4.3)
3–5 days per week	1128 (6)	22 (12)	3.5 (2.1, 6.0)	902 (6)	11 (17)	5.1 (2.3, 11.4)
6–7 days per week	704 (4)	26 (14)	5.8 (3.5, 9.7)	424 (3)	9 (14)	8.0 (3.4, 18.8)

Abbreviations: CI, Confidence interval; OR, Odds ratio; PD, Parkinson’s disease; Ref, Reference.

^a^May not add up to 20473 and 16259 due to missing values in non-motor symptoms;

^b^Adjusted for age (restricted quadratic splines with knots at 38, 46, and 55 years for men and continuous for women), and smoking status; separate models for each symptom.

Note: analysis not shown for reduced sense of smell and dream-enacting behavior for women because of small sample size.

### Number of NMS and PD

The number of NMS was strongly associated with prevalent PD (Table 5). Compared to men without NMS, the OR was 5.5 for one, 17.0 for two, 53.4 for ≥ three NMS (P_trend_ < 0.0001). Associations were stronger for PD with longer duration than shorter duration, and the difference was statistically significant (P = 0.006). We saw similar but less strong dose-response relationships among women; the OR was 4.6 for one, 6.7 for two, and 23.6 for ≥ three symptoms (P_trend_ < 0.0001). The associations appeared stronger for women with PD duration > 5 years but were not statistically different from those with shorter duration (P = 0.12). The cross-product term between sex and the number of NMS was marginally statistically significant (P_interaction_ = 0.07).

**Table 5:** Number of non-motor symptoms and Parkinson's disease in the Agricultural Health Study.

Number of NMS	No PD	All PD cases		PD diagnosis ≤ 5 years		PD > 5 years	
	N (%)	N (%)	OR (95% CI)^a^	N (%)	OR (95% CI)^a^	N (%)	OR (95% CI)^a^
Men	N = 20282	N = 191		N = 103		N = 84	
0	13332 (66)	25 (13)	Ref	16 (16)	Ref	7 (8)	Ref
1	4967 (25)	55 (29)	5.5 (3.4, 8.8)	32 (31)	4.9 (2.7, 9.0)	22 (26)	8.0 (3.4, 18.7)
2	1296 (6)	45 (24)	17.0 (10.4, 28.0)	28 (27)	16.1 (8.6, 30.1)	17 (20)	23.8 (9.8, 57.7)
≥ 3	583 (3)	66 (35)	53.4 (33.2, 86.1)	27 (26)	32.6 (17.2, 61.5)	38 (45)	116.7 (51.5, 264.7)
Women	N = 16191	N = 68		N = 38		N = 29	
0	8715 (54)	10 (15)	Ref	7 (18)	Ref	2 (7)	Ref
1	5297 (33)	29 (43)	4.6 (2.3, 9.5)	18 (47)	4.1 (1.7, 9.9)	11 (39)	8.7 (1.9, 39.4)
2	1490 (9)	12 (18)	6.7 (2.9, 15.6)	5 (13)	4.0 (1.3, 12.8)	7 (25)	19.3 (4.0, 93.1)
≥ 3	590 (4)	16 (24)	23.6 (10.7, 52.4)	8 (21)	17.1 (6.2, 47.6)	8 (29)	57.7 (12.2, 273.4)

Abbreviations: CI, Confidence intervals; NMS, Nonmotor symptoms; OR, Odds ratio; PD, Parkinson’s disease.

^a^Adjusted for age (restricted quadratic splines with knots at 38, 46, and 55 years for men and continuous for women), smoking status (current, former, and never smokers for men and never and ever smokers for women), and for other symptoms.

Note: P-value for the cross-product term between sex and number of NMS = 0.07 in the overall sample.

P-values for the cross-product terms between sex and number of NMS > 0.10 for duration-specific analysis.

Duration information missing for N = 4 for men and N = 1 for women.

## Discussion

In this cross-sectional analysis of male farmers and female spouses, we confirmed that, among the NMS evaluated, reduced sense of smell and dream-enacting behaviors had the strongest associations with prevalent PD. We further determined that the number of NMS was associated with PD in a strong dose-response manner. Although these relationships held in both men and women, in general the associations were stronger in men.

The notion that NMS and other preclinical markers of PD could be used as potential screening tools to characterize populations at higher risk for PD is being evaluated in clinical and epidemiological studies. While this idea is appealing, putting it into practice is challenging. Most NMS are common among older adults. This fact, coupled with the relatively low incidence of PD, results in very low positive predictive value of any individual NMS for PD screening in the general population. On the other hand, if these symptoms have a shared pathological basis, as suggested by the Braak hypothesis [8, 9], investigations on multiple NMS and PD may advance efforts to identify populations at risk for PD. Further, compared with motor dysfunction, NMS in PD patients are often not adequately managed and disproportionally affect patients’ qualify of life [22]. Therefore, research on NMS may also lead to a better clinical management of PD and improved quality of life.

To the best of our knowledge, only a handful of studies have examined multiple NMS simultaneously in relation to PD [11, 22–25], The Honolulu Asia Aging Study examined impaired olfaction, constipation, daytime sleepiness, and impaired executive function in relation to PD risk. PD incidence increased from 16 per 10,000 person-years for participants without any NMS to 215 for those with three or more NMS [26]. A few other studies have reported greater burden of multiple NMS in patients with early PD than in controls [25, 27, 28], and suggested that burden could be higher in certain PD subtypes [28]. Providing further support for the concept that multiple NMS are a marker of common pathogenesis, a recent study has shown that progression of RBD to neurodegenerative disorders tends to be faster in patients with olfactory loss and/or abnormal color vision than in RBD patients without these additional NMS [29].

Overall, the available evidence supports the notion that these NMS tend to cluster among PD patients; however quantitative evidence from well-designed prospective studies in the general population is needed. The current cross-sectional analysis, while subject to several limitations, may inform future longitudinal research.

Several key findings are worth mentioning. Of individual symptoms, reduced sense of smell and dream-enacting behaviors were the most important. A previous study found that olfactory function alone outperformed genetic risk scores and most other potential markers in differentiating PD cases from controls [24]. The literature also identified dream-enacting behavior as the most specific NMS for PD and other α-synucleinopathies [30]. Together with data from our study, these findings suggest that poor olfaction and RBD alone or in combination may be especially useful in effectively discriminating PD patients from controls and in identifying at-risk populations.

Second, our results suggest potential sex differences in the associations of NMS with PD. Several lines of evidence hint at sex differences in PD etiology and progression [31,32], including higher PD prevalence and earlier onset in men, milder phenotype in women, some differences in motor features, and greater NMS fluctuation in women with disease progression. In the same manner, a handful of recent studies have also suggested potential sex- differences in NMS presentations in PD patients [11, 31–36]; for example, of the NMS we examined, previous studies have identified poor olfaction [11, 33–38], excessive daytime sleepiness [2,35,38], RBD [37] were more common in men, whereas anxiety [11,36], depression [36,38,39], and constipation [38] were more common in women. However, most of these studies were limited in that they did not have a comparison control group [33,35,38], or they presented only sex-specific NMS frequencies [34,36]. Of note, one study reported sex-specific NMS combinations that could differentiate *de novo* PD cases from healthy controls [11]. In our analysis, ORs for associations between loss of sense of smell and PD were much higher for men than for women. Interestingly, in both PD patients and the general older adult populations, men performed worse on olfaction tests than women [11, 40]. Future investigations should further investigate this potential sex difference in PD presentation as well as in PD development and progression.

Third, our data showed dose-response relationships for several symptoms for which we had information on frequency. For example, we found a dose-response relationship between the frequency of dream-enacting behavior and PD; interestingly even infrequent dream-enacting behavior (i.e., < 3 times in life or < 1 time per month) was associated with 4–5 times higher odds of having PD. Dose-response relationships were also suggested for bowel movement frequency and daytime sleepiness with PD, consistent with prior studies [4, 19]. Future studies might benefit from considering symptom severity, rather than only the presence or absence of individual NMS.

Strengths of our study include the relatively large sample size and preplanned analyses of multiple NMS and of potential sex differences. Our study also has limitations. First, it is a cross-sectional study, so it is hard to draw conclusions about temporality. However, significant associations were seen even among cases diagnosed 5 or fewer years prior to Phase 4, suggesting that associations could be an early manifestation or even predate clinical PD onset. Only a prospective study, however, can fully address this question. Second, NMS was self-reported and bias due to differential reporting between PD cases and individuals without PD is possible and may in part account for the strong associations we observed. Still, the magnitudes of associations identified in our study are in the range of estimates from other studies that measured individual NMS with objective tests or structured questionnaires [11, 25]. Only a prospective study with objective measurements of NMS, however, can fully address this question. Third, we chose to screen for NMS using simple questions that are practical in large population-based epidemiological studies. Although these questions have been validated or used in previous studies, some misclassification is likely. Fourth, we relied on self-reported physician diagnosed PD to identify PD cases; we therefore might have inadvertently missed or misclassified some cases. However, previous analyses in this population showed reasonably high agreement (84%) between self-report and expert adjudication[13]. Further, we replicated known associations of PD with age and smoking status, providing indirect support for the validity of case ascertainment. Fifth, most PD cases were prevalent and likely using medications which might have affected the presence of NMS. For example, it is possible that the associations with depression and anxiety have been underestimated and that for excessive daytime sleepiness overestimated, as dopaminergic medications may improve depression and anxiety, but exacerbate excessive daytime sleepiness [41]. Unfortunately, the lack of detailed data on medications for PD and these affective symptoms did not allow us to investigate the roles of these medications on NMS of PD, and how they would affect the current analyses. Sixth, our findings suggest some sex-specific NMS-PD associations, which may in part due to differences in occupational exposures such as pesticide use. Seventh, there is potential for selection bias if the presence of NMS or PD diagnosis affected Phase 4 participation. Lastly, as our study participants were mainly farmers, the results may not be generalizable to other populations.

In summary, we found that the number of NMS was associated with PD in a dose-response manner in a cohort of male farmers and their female spouses. Our findings are consistent with the idea that assessing multiple NMS may help in PD risk characterization and further suggest that such risk characterization approaches should consider sex differences in NMS-PD associations. These findings should be further investigated in population-based prospective studies.

## Supporting information

S1 Fig Flowchart depicting selection of study participants. (DOCX)

S1 Table Questions for non-motor symptoms in the Agricultural Health Study Phase 4 interview. (DOCX)
