# Supplementary material for: Nonmotor symptoms and Parkinson disease in United States farmers and spouses
Source: PLoS One. 2017 Sep 27;12(9):e0185510. doi: 10.1371/journal.pone.0185510 (PMC5617219; doi:10.1371/journal.pone.0185510)
Supplement: S1 Fig — (DOCX) [file pone.0185510.s001.docx]

**S1 Fig 1: Flowchart depicting selection of study participants**


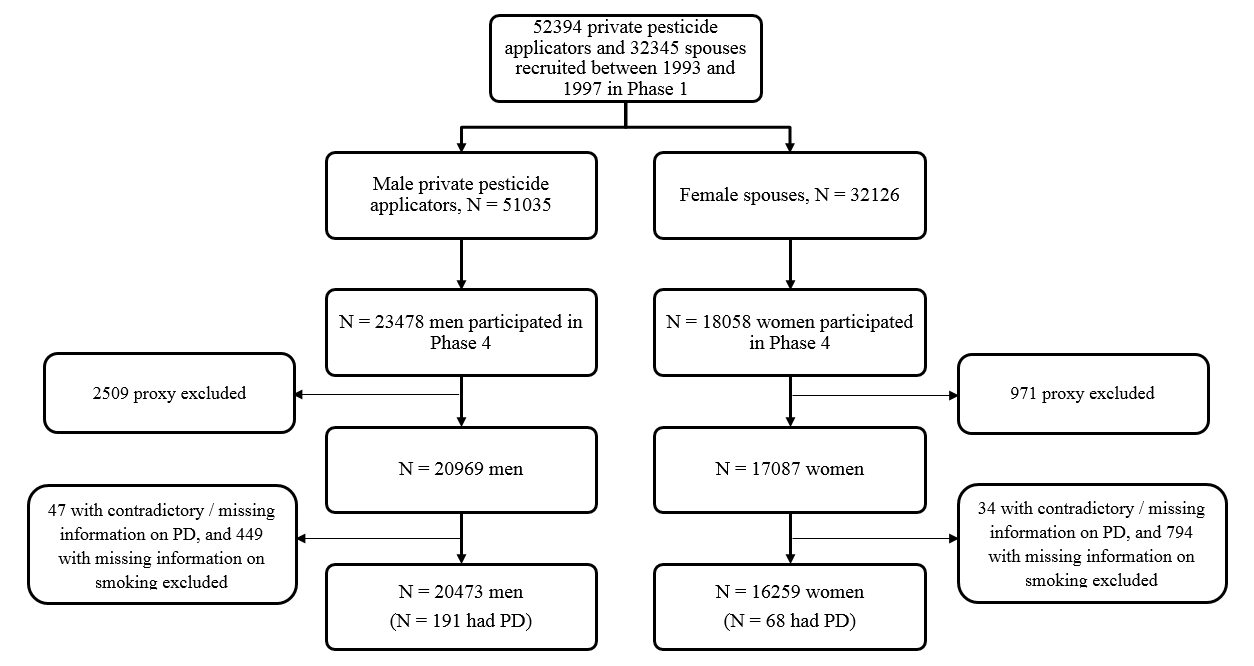


Abbreviations: PD, Parkinson’s disease
