# Supplementary material for: Nonmotor symptoms and Parkinson disease in United States farmers and spouses
Source: PLoS One. 2017 Sep 27;12(9):e0185510. doi: 10.1371/journal.pone.0185510 (PMC5617219; doi:10.1371/journal.pone.0185510)
Supplement: S1 Table — (DOCX) [file pone.0185510.s002.docx]

**S1 Table 1: Questions for non-motor symptoms in the Agricultural Health Study Phase 4 interview.**

| Non-motor symptoms | Question^a^ | Response choices | Definition^b^ |
| --- | --- | --- | --- |
| Reduced sense of smell | Do you suffer from a loss of sense of smell, or significantly decreased sense of smell? | Yes/ No | Yes |
| Dream-enacting behavior | Have you ever been told, or suspected yourself, that you seem to "act out your dreams" while sleeping? For example, punching or flailing arms in the air, shouting, or screaming while asleep. | Yes/ No | Yes |
|  | How often have you "acted out your dreams"? | < 3 times in your life < Once a month 1–3 times per month Once a week > Once per week Don't know |  |
| Excessive daytime sleepiness | How often do you feel sleepy most of the day? | Never < 1 day per month 1–3 days per month 1–2 days per week 3–5 days per week 6–7 days per week | 6–7 days per week |
| Infrequent bowel movement | Have you ever taken any over-the-counter or prescribed medicines to help with bowel movements? Do not include medications taken only a few times a year. | Yes/ No | Ever taken medication, or bowel movement frequency of ≤ 3-4 times/ week |
|  | Typically, how often do you have bowel movements? | ≥ 2 times per day Once per day 5–6 times per week 3–4 times per week  < 3 times per week |  |

| Depressive symptoms | | Are you currently taking any prescribed medicines for depression? | Yes/ No | | Currently taking medicines for depression, or total PHQ-2 score ≥ 3 | |
| --- | --- | --- | --- | --- | --- | --- |
|  |  | Over the last two weeks, how often have you been bothered by^c,d^  a) having little interest or pleasure in doing thing  b) feeling down, depressed, or hopeless | Not at all  Several days  More than half the days  Nearly every day | |  |  |
| Anxiety symptoms | | Over the last two weeks, how often have you been bothered by^c,e^ a) feeling nervous, anxious, or on edge b) not being able to stop or control worrying | Not at all Several days More than half the days Nearly every day | | Total GAD score ≥ 3 | |
| ^a^Questions were asked in Phase 4 of the Agricultural Health Study. | | |  |  |  |  |
| ^b^Participants were considered having NMS based on response from single or multiple questions. | | |  |  |  |  |
| ^c^Responses – not at all, several days, more than half the days, and nearly every day were assigned a score of 0, 1, 2, and 3, respectively; scores from two responses were summed to obtain total score for an individual.  ^d^Patient health questionnaire – 2 (PHQ-2).  ^e^Generalized anxiety disorder questionnaire – 2 (GAD). | | | | |  |  |
